# Supplementary material for: Causal evidence for the adaptive benefits of social foraging in the wild
Source: Commun Biol. 2021 Jan 20;4:94. doi: 10.1038/s42003-020-01597-7 (PMC7817680; doi:10.1038/s42003-020-01597-7)
Supplement: Supplementary file 3 — Reporting Summary [file 42003_2020_1597_MOESM3_ESM.pdf]

nature research

Corresponding author(s): Lysanne Snijders

Last updated by author(s): Dec 8, 2020

Reporting Summary

Nature Research wishes to improve the reproducibility of the work that we publish. This form provides structure for consistency and transparency in reporting. For further information on Nature Research policies, see our Editorial Policies and the Editorial Policy Checklist.

Statistics

For all statistical analyses, confirm that the following items are present in the figure legend, table legend, main text, or Methods section.

n/a	Confirmed	
☐	☒	The exact sample size (*n*) for each experimental group/condition, given as a discrete number and unit of measurement
☐	☒	A statement on whether measurements were taken from distinct samples or whether the same sample was measured repeatedly
☐	☒	The statistical test(s) used AND whether they are one- or two-sided<br>*Only common tests should be described solely by name; describe more complex techniques in the Methods section.*
☐	☒	A description of all covariates tested
☐	☒	A description of any assumptions or corrections, such as tests of normality and adjustment for multiple comparisons
☐	☒	A full description of the statistical parameters including central tendency (e.g. means) or other basic estimates (e.g. regression coefficient) AND variation (e.g. standard deviation) or associated estimates of uncertainty (e.g. confidence intervals)
☐	☒	For null hypothesis testing, the test statistic (e.g. F, t, r) with confidence intervals, effect sizes, degrees of freedom and P value noted<br>*Give P values as exact values whenever suitable.*
☒	☐	For Bayesian analysis, information on the choice of priors and Markov chain Monte Carlo settings
☐	☒	For hierarchical and complex designs, identification of the appropriate level for tests and full reporting of outcomes
☐	☒	Estimates of effect sizes (e.g. Cohen's d, Pearson's r), indicating how they were calculated

Our web collection on statistics for biologists contains articles on many of the points above.

Software and code

Policy information about availability of computer code

Data collection	Behaviour during the experimental trials was quantified using BORIS v 7.5, a free open-source event-logging software. More information about the program can be found in: Friard O, Gamba M. 2016 BORIS: a free, versatile open-source event-logging software for video/audio coding and live observations. Methods Ecol. Evol. 7, 1325–1330. (doi:10.1111/2041-210X.12584)
Data analysis	All data analyses were conducted in R version 4.0.2. Names of specific packages are reported in the manuscript. The code (and associated datasets) to reproduce all the findings reported in the manuscript is available in the Open Science Framework repository, https://osf.io/csajg

For manuscripts utilizing custom algorithms or software that are central to the research but not yet described in published literature, software must be made available to editors and reviewers. We strongly encourage code deposition in a community repository (e.g. GitHub). See the Nature Research guidelines for submitting code & software for further information.

Data

Policy information about availability of data

All manuscripts must include a data availability statement. This statement should provide the following information, where applicable:

- Accession codes, unique identifiers, or web links for publicly available datasets
- A list of figures that have associated raw data
- A description of any restrictions on data availability

All the data and code generated and analyzed during the current study are available in the Open Science Framework repository, https://osf.io/csajg

Field-specific reporting

Please select the one below that is the best fit for your research. If you are not sure, read the appropriate sections before making your selection.

- [x] Life sciences
- [ ] Behavioural & social sciences
- [ ] Ecological, evolutionary & environmental sciences

For a reference copy of the document with all sections, see nature.com/documents/nr-reporting-summary-flat.pdf

Life sciences study design

All studies must disclose on these points even when the disclosure is negative.

Sample size	No initial sample size calculation was performed for the experiment as there was no a-priori indication of effect size. This was the first study to experimentally manipulate number of conspecifics, including solitary individuals, to evaluate foraging success in wild guppies (or other vertebrates). The number of trials per batch was informed by two previous studies:<br>- Snijders L, Kurvers RHJM, Krause S, Ramnarine IW, Krause J. 2018 Individual- and population-level drivers of consistent foraging success across environments. Nat. Ecol. Evol. 2, 1610–1618. (doi:10.1101/260604)<br>- Snijders L, Kurvers RHJM, Krause S, Tump AN, Ramnarine IW, Krause J. 2019 Females facilitate male food patch discovery in a wild fish population. J. Anim. Ecol. 88, 1950–1960. (doi:10.1111/1365-2656.13086)
Data exclusions	Data from failed or bad video recordings were excluded for a number of the analyses. All exclusions, including the number of exclusions and their criteria are reported in the Methods section of the manuscript.
Replication	No attempt of replication of the reported study as a whole has been made, yet the study itself included replicates for sex-composition and number of conspecifics. In addition all experiments and treatment levels were replicated across seven different natural pools, using 84 unique batches (39 female batches, 35 male batches, 10 control batches). Exact numbers per treatment level are reported in Supplementary Table 1 and 2.
Randomization	Individuals originated from the same location as their batch members to maximize familiarity among the fish. Assignment to treatment-level and pool was done according to an a-priori determined daily schedule. Location order of food trials was randomized.
Blinding	Blinding was not possible in this study as both the number of fish as the sex of fish are impossible to conceal during observation. The video observers were, however, not informed about the underlying hypotheses.

Reporting for specific materials, systems and methods

We require information from authors about some types of materials, experimental systems and methods used in many studies. Here, indicate whether each material, system or method listed is relevant to your study. If you are not sure if a list item applies to your research, read the appropriate section before selecting a response.

Materials & experimental systems

n/a	Involved in the study	
x		Antibodies
x		Eukaryotic cell lines
x		Palaeontology and archaeology
	x	Animals and other organisms
x		Human research participants
x		Clinical data
x		Dual use research of concern

Methods

n/a	Involved in the study	
x		ChIP-seq
x		Flow cytometry
x		MRI-based neuroimaging

Animals and other organisms

Policy information about studies involving animals; ARRIVE guidelines recommended for reporting animal research

Laboratory animals	The study did not involve laboratory animals
Wild animals	We assigned 392 study subjects of which 14% escaped prior to data collection (41 out of 235 females and 14 out of 157 males). The experiment thus included 337 experimental subjects (194 females and 143 males), spread over 84 batches (39 female batches, 35 male batches, 10 control batches; see Supplementary Table 1 and 2 for further details). Animals were caught using dip nets and transported using small semi-transparent containers with an opaque lid and including water from their stream and a leaf under which the fish could hide. The guppies were caught and marked using fluorescent elastomer (Croft et al. 2004). Fish were given individual identity marks by injecting different colours of visible implant fluorescent elastomer (VIE) in two out of six dorsal positions. For this procedure we followed Croft et al. (2003 & 2004). Fish length was measured briefly (max 5 seconds) using a measuring line, while avoiding skin to skin contact. Fish from different source pools were kept for a maximum of 2 hours in separate plastic containers during the marking procedure after which they were released in their respective experimental pools. The fish were left overnight in

their experimental pools to acclimatise to their new surroundings. Foraging trials took place the next day. After finishing the foraging trials, we released subjects further downstream (to avoid recapture) on the same day.
- Croft DP, Arrowsmith BJ, Bielby J, Skinner K, White E, Couzin ID, Magurran AE, Ramnarine I, Krause J. 2003 Mechanisms underlying shoal composition in the Trinidadian guppy, Poecilia reticulata. Oikos 100, 429–438. (doi:10.1034/j.1600-0706.2003.12023.x)
- Croft DP, Krause J, James R. 2004 Social networks in the guppy (Poecilia reticulata). Proc. R. Soc. Lond. B 271, S516–S519. (doi:10.1098/rsbl.2004.0206)

Field-collected samples	The study did not involve samples collected from the field
Ethics oversight	We performed all research in accordance with the ‘Basic Principles Governing the Use of Live Animals and Endangered Species in Research at the University of the West Indies’ as part of the ‘Policy and Procedures on Research Ethics’ of the University of the West Indies Committee on Research Ethics.

Note that full information on the approval of the study protocol must also be provided in the manuscript.
